# A principled ethical approach to intersex paediatric surgeries

**DOI:** 10.1186/s12910-020-00550-x

**Published:** 2020-10-29

**Authors:** Kevin G. Behrens

**Affiliations:** Steve Biko Centre for Bioethics, School of Clinical Medicine, Faculty of Health Sciences, University of the Witwatersrand, Phillip V. Tobias Health Sciences Building, 29 Princess of Wales Terrace, Parktown, Johannesburg, 2193 South Africa

**Keywords:** Intersex, Intersex surgery, Intersex paediatric surgery, Intersex ethics

## Abstract

**Background:**

Surgery for intersex infants should be delayed until individuals are able to decide for themselves, except where it is a medical necessity. In an ideal world, this single principle would suffice and such surgeries could be totally prohibited. Unfortunately, the world is not perfect, and, in some places, intersex neonates are at risk of being abandoned, mutilated or even killed. As long as intersex persons are at such high risk in some places, any ethical guidelines for intersex surgeries will need to take these extreme risks of harm into account.

**Main text:**

I therefore argue for five basic principles that ought to inform ethics guidelines for surgical interventions in intersex children, specifically in contexts in which such children are at risk of significant harm. What I set out to come up with is a set of principles that do not completely prohibit surgery, but only allow it where a strong case can be made for its necessity, in the best interests of the child, and where there is some kind of oversight to prevent misuse. The first principle is that interventions as drastic as these surgeries should only be performed when there is strong evidence that they are beneficial and not harmful. The second principle is that in surgeries should normally only be performed in cases of true medical necessity. Principle three is that surgeries should normally be delayed until such time as the intersex person is mature enough to assent to treatment or decide against it. Principle four is that the conventional ethical requirements regarding truth telling apply equally to intersex children as to anyone else. The final principle is that where physicians or parents think that surgery is in the best interests of the child, the burden of proof lies with them.

**Conclusion:**

It is hoped that these principles might help medical teams and parents make better decisions about intersex surgeries on children, and they would make such surgeries very rare indeed, if they happen at all.

## Background

On 24 January 2018, a South African newspaper, the *Mail and Guardian*, posted an article online. It was entitled ‘Intersex babies killed at birth because they are bad omens’. In it the author describes an incident in a rural South African village in which a traditional healer, assisting with the delivery of a baby, witnessed how the baby was killed by family members when it was discovered that it had been born with ambiguous genitalia. They decided to lie to the mother and tell her that the child was stillborn. The author points out that this is an example of many cases of intersex infanticide that occur because of a belief that ‘intersex babies are bad omens. They are seen as a sign of witchcraft and a curse on the family and the community as a whole’ [1].

While we do not have reliable statistical data for how often intersex infanticide takes place in South Africa, there is sufficient evidence to know that it does occur. In an informal study conducted by Tunchio Teriso in a rural area of South Africa, 88 out of the 90 traditional midwives and birth attendees interviewed owned up to having killed babies born with ambiguous genitalia [1, 2]. Furthermore, we know that, in a context in which an intersex person is sometimes believed to be a bad omen, a curse, or a sign of punishment, witchcraft, or the displeasure of ancestors, intersex children are exceptionally vulnerable to social stigma, rejection and even physical violence [1, 2]. It is against this contextual backdrop that I seek, in this paper, to develop a set of principles to guide medical teams and parents to make ethically justified decisions about intersex paediatric surgeries.

While this article has arisen out of the situation in rural South Africa described above, it is of relevance in other contexts, too. It is likely that if intersex babies are killed in South Africa, they are also killed in other countries on the continent, and indeed in other parts of the world, where similar beliefs about intersex children prevail. Intersex infanticide or attempted infanticide has been reported from Uganda [3], Kenya [4] and China [5]. It is likely that the most vulnerable intersex infants are those born in rural communities, where births take place in private homes and out of sight of authorities. Furthermore, globally, intersex infants are at risk of being discarded, abandoned, neglected or mutilated [6]. Intersex individuals are also vulnerable to violence and even murder after infancy and right through into adulthood. What all of this suggests is that there is a need for an approach to early infant intersex surgeries that is cognisant of the very real threat to life and limb of intersex individuals.

The first talk I gave on this subject was to staff and students of the Philosophy department at a neighbouring university in Johannesburg. I took a hard line, arguing for law reform to completely prohibit medically unnecessary intersex surgeries before an age where intersex persons can give consent or at least express their wishes. During question time, I was challenged on this by one of the academics in the audience. He asked me whether I had considered the possibility that in the South African context, prohibiting surgeries completely might have the unintended consequence of leading to even more intersex infanticide or abandonment. Perhaps, he said, the option of surgery could potentially save lives, and removing the option could have disastrous consequences.

Another person asked whether—in communities in which intersex persons are so feared—surgery might not sometimes be necessary to prevent a child from experiencing terrible ostracization, rejection, violent assault, and even murder. Of course, I defended my position by saying that we should not give in to prejudice and ignorance, and we should work on the root of problem: changing beliefs and attitudes in the community, so that intersex people are welcomed and not rejected. But, I left the talk feeling rather perplexed and uncertain of my position. How would I feel if the total prohibition of child surgeries did have the effect of a significant rise in infanticide or abandonment, or if worried parents resorted to back street surgeries, with the predictable, harmful consequences? Changing beliefs and entrenched cultural practices takes time. We would be foolish to think that that it would not take decades for even well-conceived and funded education programmes to be successful in effecting such change. So, my interlocuters had a point. I would need to carefully consider the potentially very harmful unintended consequences of simple prohibition. It is for this reason that I here adopt a slightly less rigid approach that would make surgical procedures a rare exception only to be used in cases where they might be necessary to protect an intersex person from serious harm.

In this paper, my aim is not to argue for legal reform, but rather to address the problem of early intersex surgeries^1^ by seeking to influence the decisions and actions mainly of health care professionals. Partly this is because I am employed as a teacher of bioethics to such professionals. However, I am also mindful of the very significant role health professionals play in the decisions made by parents regarding the treatment of their intersex children. Parents frequently rely on their counsel as medical experts, and their opinions are very influential. While it is parents who must give consent for treatment and surgery, it is often the physicians’ views that swing the outcome [7, 8], even if (in some cases) it is only in conceding to parental choices where these are not in the best interests of the child. Thus, I have chosen to essentially target health professionals and, through them, the parents of intersex children.

I have also chosen to focus on health professionals because it is the case that resorting to early surgeries remains a common response to intersex births in my home country, South Africa, as well as in many other countries. With what is already a comparatively high incidence of intersex births [1], South Africa has no official professional ethical guidelines for medical specialists on such surgeries and is not party to the Chicago Consensus [9], the one multinational set of professional guidelines that does exist. This results in a situation in which surgery is the default option, seriously affecting large numbers of intersex children born in the country [10]. While it is difficult to find data on the prevalence of infant genital ‘normalisation’ surgeries in other parts of the world, where intersex individuals are at significant risk of harm, it is clear that there are still many countries in which early surgery remains the preferred approach to intersex births. What is required, in places where such surgeries are available, are some ethical guidelines aimed at ensuring that surgery does not remain the default option, and that it is only chosen in cases where it can be robustly defended, ethically. This paper is intended to provide some basic principles to guide health professional bodies to develop appropriate guidelines on infant intersex surgeries. I am very aware that this can, at best, be one component of a much broader and multi-faceted strategy that is necessary to address all of the issues around the human rights and well-being of the intersex community, including education, advocacy, legal reform, social change and even moves toward a global ban on surgeries. However, I hope that by getting through to those who are most involved in the decisions about surgery, the health professionals and parents, surgeries will become at best very rare and certainly no longer the default choice.

I propose five basic principles that ought to be used as the scaffolding for a set of ethics guidelines for surgical interventions in intersex children, specifically in contexts in which intersex children are at risk of significant harm. What I set out to come up with is a set of principles that does not completely prohibit surgery, but only allows it where a strong case can be made for its necessity, in the best interests of the child, and where there is some kind of oversight to prevent misuse. I believe these principles are congruent with the Malta Declaration developed by the International Intersex Forum [11].^2^

## Principle 1: interventions as drastic as these surgeries should only be performed when there is strong evidence that they are, all things considered, beneficial and not harmful

One might think that this goes without saying. However, whilst it has often been claimed that genital ‘normalising’ surgeries^3^ that are not a strict medical necessity are beneficial, there is a paucity of evidence to justify such claims. These surgeries first began to be done at the Johns Hopkins Hospital in Baltimore in the 1950s, and spread around the world, soon becoming the global standard of care. Primarily grounded in the work of the psychologist, John Money, they were justified on two main assumptions, the first being that gender identity is more a matter of nurture than nature. So long as the child had surgery to ‘normalise’ the appearance of the genitals, the child would grow up to identify with the gender it was raised as [7, 12]. The second was the belief that it was in the best interests of the intersex child to be raised as ‘normally’ as possible. Surgery would help the child to appear more ‘normal’ and ‘fit in’ better socially [13, 14]. We now know that both of these assumptions turned out to be false.

The first claim—that it was possible to socialise a child into a chosen gender identity—was shown to be false very soon. Money frequently defended his claim by making reference to a case that was to become famous. He had been approached for advice on how to manage a situation in which a botched circumcision on an 8 month old identical twin boy had caused the child to lose his penis. Money advised the parents to obtain surgery to remove the boy’s testicles and reconstruct the external genitalia to resemble the typical genitalia of a girl. Furthermore, he recommended that the child be raised as girl, and that his medical history should be hidden from him. Convinced that gender identity could be altered by such socialisation, Money believed that it would be better for the child to undergo the surgery and be raised as a girl. He believed this would be better than the alternative ‘to raise him as a boy with an inadequate penis’ which would purportedly cause ‘the child [to] suffer severe psychological trauma’ [7]. Despite the fact that the child in this case was not born intersex, at the time, the case was lauded as evidence that Money was right about how best to treat intersex infants. Julie Greenberg describes how this case was received in the context of the time as follows:

This ‘male turned into a female’ case made headlines. Because the doctors involved in the treatment reported that the child and the parents had successfully adapted to the sex/gender alteration, sociology, psychology, and women’s studies texts were rewritten to argue,’[t]his dramatic case... provides strong support... that conventional patterns of masculine and feminine behaviour can be altered. It also casts doubt on the theory that major sex differences, psychological as well as anatomical, are immutably set by the genes at conception.’ [13]

The purported success of this case became the subject of much debate some years later, in 1997, when Milton Diamond and Keith Sigmundson published an article that told the story of what had become of the twin boy who had been raised as a girl. Contrary to Money’s claims that the child had self-identified as a girl, he had, in fact, always preferred typically male toys and behaviours, even preferring to urinate standing up. At the age of 14, he confided in a doctor that he identified as a boy and wished to live as boy. He then began the process of re-transitioning back to male [13]. He was to live the rest of his life as a male, even marrying a woman and adopting her children, before his death by suicide in 2004 at the age of 38 [14].

The failure to socialise a child into a chosen gender identity after reassignment surgery in this one case is insufficient evidence, on its own, to refute Money’s original claims. More evidence was soon to follow, however. Greenberg cites a presentation given by William Reiner, a urologist and psychiatrist at Johns Hopkins Hospital, at a Paediatric Endocrine Society meeting in 2000. Reiner reported on preliminary findings of a study of 27 infant boys born without penises. Of these, 25 had undergone sex re-assignment surgery and had been raised as girls. Of these, only 14 ended up identifying as boys. Furthermore, the two infants who had not undergone surgery and were raised as boys were ‘better adjusted’ than the others [13]. Dolgin writes:

research has failed to demonstrate that early surgery to re-shape an intersex child’s genitals, accompanied by socialization within the assigned gender, results in a better or ‘more typical’ childhood. To the contrary, surgery to conform the appearance of genitalia to a gender selected by doctors and/or parents early in a child’s life is likely to result in psychological difficulties that affect the child and the adult that child will become [14].

Far too many intersex adults who were raised as one gender or the other, after genital ‘normalisation’ surgery, turned out not to identify with the gender that had been assigned to them [15–17]. Of course, the knowledge we now have about gender identity shows that it is much more complex than we used to think it was, as the very existence of transgender people demonstrates. Furthermore, the whole idea was originally grounded in the work of Money, the underlying assumptions of which have been discredited. By now, the idea that we can simply socialise a child into a chosen gender has been thoroughly debunked and any health professional still making that claim opens themselves to the accusation of being ignorant or wilfully ignoring the evidence. Yet, these surgeries continue. Most often, this is because the health professionals still hold to the truth of the second assumption, that surgery is in the best interests of the child. This is a claim about the psycho-social well-being of the intersex person. Returning to my principle, I have stated that because these interventions are so drastic, they should only be performed where there is evidence that they are, all things considered, beneficial and not harmful. There is plenty of evidence that these surgeries cause many kinds of harm. Greenberg writes that some intersex activists and experts

believe that the traditional model results in stigma and trauma. Because of the emphasis on ‘normalizing’ the infant’s genitalia, parents will experience guilt and shame over giving birth to an ‘abnormal’ baby and the intersex patient will experience a sense of rejection. They question the traditional assumption that concealing or downplaying the existence of the intersex condition will help the family lead a ‘normal’ life [13].

Cresti describes some of the consequences of early surgeries as ‘pain, lifelong depression, incontinence, and scarring’ and points out that

another objection is that early surgery is ‘worthless mutilation’ that causes damage to individuals who have not chosen to be subjected to those interventions. Indeed, those interventions reduce sexual pleasure in many cases…, impose a sex that might not coincide with future gender identity, and in most cases are irreversible. Furthermore, outcome studies are scarce and surgical outcomes are uncertain [15].

Greenberg provides a comprehensive account of the harms that intersex persons who have had early surgery experience:

During the 1990s, a number of intersex activist groups also began to question the standard protocol for treating intersexuality. Because genital surgery may result in a loss of reproductive capacity, a loss of erotic response, genital pain or discomfort, infections, scarring, urinary incontinence, and genitalia that are not cosmetically acceptable, these groups believe that such surgery should not be performed without the informed consent of the intersex patient. In addition, they maintain that the current treatment protocol exacerbates an intersexual’s sense of shame by reinforcing cultural norms of sexual abnormality [13].

I am not aware of any studies or other actual evidence the supports the idea that it is, in fact, beneficial to the psycho-social well-being of intersex people when they are subjected to early surgery. In the absence of evidence, we should always make decisions based on what we actually do know [13]. So, until research is done showing that there are such benefits, we are morally obliged not to perform such drastic surgeries.

## Principle 2: surgeries should normally only be performed on intersex infants in cases of true medical necessity

If my claims in the previous section are right, if a child’s gender identity cannot be fixed just by rearing, and if it is not at all clear that the psycho-social consequences of infant genital ‘normalisation’ are more beneficial than harmful, then there is, ordinarily, no justification for proceeding with these surgeries, except where there is a medical necessity.^4^

There are some cases in which intersex conditions may require immediate surgical intervention, such as when there is malignant tissue that needs to be removed or when an opening for urination needs to be created [8]. Of course, there is likely to be disagreement among experts over what conditions do require early surgical intervention. While total consensus might elude us, medical profession bodies should be able to come up with satisfactory guidelines for best practice in this regard. The point of my principle is to allow for paediatric intersex surgery in the rare cases where it is arguably a medical necessity, while preventing surgeries for any other reasons.

It should be noted that this principle deals with surgical interventions only. There may be other serious or even life-threatening conditions affecting intersex infants that require immediate medical attention (for example, congenital adrenal hyperplasia can be fatal in the early stages of infancy if not treated with steroids) [12]. Since the focus of this article is only on paediatric intersex surgeries, these will not be discussed further here.

Setting aside definitional difficulties, I will simply rely on solid ethical precedent and the generally accepted moral intuition that we ought not to perform unnecessary surgeries, certainly not when the patient has not expressly requested such of their own accord. I will rest my case with this for now.

## Principle 3: surgeries should normally be delayed until such time as the intersex person is mature enough to assent to treatment or decide against it

The exceptionalism that routinely applies in these cases is quite baffling: where else would we routinely override the conventional ethical requirements for age-appropriate assent or informed consent for elective surgeries with no medical necessity and where the consequences could be very harmful to the patient? It is trite to say that is established ethical practice not to perform such surgeries without the agreement of the person having the surgery. In the case of children, it is generally regarded as both legally and ethically permissible for parents or legal guardians to consent to treatment on behalf of minors who are not capable of giving informed consent. However, this concession is intended to allow for medically necessary or urgent interventions, and in many jurisdictions is subject to the principle that it is only permissible if done in the ‘best interest of the child’^5^ [12, 13, 15].

Cresti, Nave and Lala provide a cogent account of the ethical limitations that apply to parental consent. They write:

In the case of intersex newborns, there is no doubt about their inability to make their own decisions. Some solid moral reasons justify the attribution of decision-making ownership to the parents of minors… Such parental decision-making ownership finds a limit in the obligation not to harm, and this harm can also include any impact on the ability of the individual to exercise their autonomy in future. Their interpretation of beneficence prevails on the other interpretations, but it cannot lead to the presumption of making personal and permanent decisions better than could the intersex person herself. Surgical sex assignment leads to irreversible body modifications, body perception, and functionality, and clinical and pathological reasons do not justify this. Only individuals whose body undergoes such treatments should give informed consent to these practices [15].

Cresti et al. are quite right in asserting that parental consent to treatment cannot be justifiable if it causes harm to the child.^6^ I have already given an account of the many different kinds of serious harms such surgeries can cause. Yet, critics might argue that not performing genital ‘normalising’ surgery at an early age may cause even more weighty harms—primarily of a psycho-social nature. When discussing my first proposed principle, I referenced much evidence that calls this claim into question. To a large extent, this is a question of fact, and empirical evidence should be able to provide us with a clear answer. Unfortunately, there have not been enough focussed and comprehensive longitudinal studies on intersex persons who underwent surgery at a young age for us to have a clear picture of their well-being over time. But, we have enough evidence to suggest that many of them experience severely harmful effects [12–17]. Thus it is both prudent and ethically necessary to not subject infants to such risk of harm without their assent or consent, in all cases where a delay in making the decision on whether to proceed with surgery is possible.

Studies are even more rare regarding the long-term well-being of intersex infants who were not subjected to early surgeries. This is likely the case for a number of reasons. Firstly, from the 1950s until fairly recently, surgery was the standard treatment approach in most parts of the world, leaving few untreated persons to include in studies. Secondly, possibly because of embarrassment or fear of social exclusion, intersex individuals who have managed to get through life without being identified as intersex may not be inclined to participate in research. Be that as it may, the one very comprehensive study on the well-being of intersex individuals who had not undergone surgery was done, paradoxically, by John Money some years before he began to promote his assertion that gender identity was mutable and more a matter of nurture than nature. For his Ph.D. thesis at Harvard University in 1951, Money reviewed the cases of some 250 intersex individuals who had not received surgical intervention as infants. In particular, Money focussed on the experiences of children who naturally developed to have genitals of the sex opposite to the sex of their rearing. It was his initial expectation that such individuals would struggle to adjust to normal life because of their obvious psycho-sexual issues. He reported that he was amazed to discover that even the most ambisexual of these intersex individuals showed no increased incidence of functional psychoses, and managed to cope with the tasks of ordinary living: holding down a job, earning a salary and getting an education [7]. The study included in-depth interviews with 10 individuals who did not receive surgical or hormonal treatment until they were old enough to decide for themselves. They came across as remarkably well-adjusted, resilient, confident and sometimes even optimistic. Colapinto writes: ‘Their lives only strengthened the investigator’s impression that the condition of the genitalia plays a strikingly insignificant part in the way a person develops a stable and healthy gender identity, not to mention a secure and confident self-image’ [7].

It is unclear what caused Money to abandon the evidence of his own PhD research. However, some 4 year later, he began publishing papers on intersex infants in which he promoted early ‘normalisation’ surgeries to align with a chosen gender of rearing as being in the best interest of these children. What his exhaustive PhD study does suggest, in the end, is that delaying surgeries does not necessarily result in any real harm for most individuals. Since it has already been shown that performing these surgeries does result in harms for at least a significant proportion of children, it is clear that the more ethically justified action would be to delay surgeries.

Cresti et al. also make the provocative, but plausible, claim that one of the harms caused by early surgeries arises out of the fact that they rob the individual of their autonomy in the future [15]. Many of these surgical interventions are partially and even fully irreversible [8, 12], and early surgeries often impose severe limitations on the medical and operative options that would still be available to the patient later in life. As Cresti et al. suggest, what is stake in these cases are matters that are extremely personal and ‘life-altering’, and that only the individuals themselves can decide how best to integrate their ‘body and personal and social identity’ [15]. They go on: ‘The incompetence of these minors is constitutive, provisional, and destined to be replaced by full decision-making and self-determination capacities. Identity and bodily integrity of intersex infants must, therefore, be defended from surgical or other assaults until they can decide for themselves’ [15].

Establishing an optimal age for when intersex children will be ready to make decisions about their gender expression and whether to undergo surgery or not is not an easy task. It is beyond the scope of this paper to discuss this here. But, it is important that this issue be given careful consideration. While infant surgeries should not be allowed, it might be too late to wait for adulthood before allowing those children who feel ready to do so to decide what they want. Hormonal treatment before the onset of puberty could go a long way to easing any transition and surgery later. As Crestio et al. write: ‘The ability of individuals to make decisions according to their values and beliefs should be able to be exercised before their bodies and their developing sexual and gender identity is irreversibly compromised’ [15]. What is clear is that the principle of delaying surgeries until children are able to make their own choices holds.

## Principle 4: conventional ethical requirements regarding veracity/truth-telling apply equally to intersex children as to anyone else

This principle is important because deception and hiding the truth have long been commonplace in how intersex children have been treated [7, 8]. In her account of what she calls the ‘current dominant medical practice’ (of performing early ‘normalisation’ surgeries), Greenberg identifies some deception or withholding of information as being almost a necessary condition for the practice to be successful: ‘This model emphasizes the need for a clear and unambiguous gender identification. To achieve this goal, the child should receive surgery and the parents and the intersex child may benefit by being told less than the whole truth about the nature of the condition’ [13]. Dickens writes: ‘Predetermining children’s futures by such interventions is also liable to require continuing deception regarding their biological and/or genetic inheritance, contrary to ethical expectations of truth telling and legal requirements of informed consent to treatment’ [12].

In other medical contexts, deception or withholding of the truth from patients would be taken to be obviously unethical. Yet, the personal experience of many intersex individuals who have been subjected to early surgeries was that they were often lied to or not fully informed about their medical histories [8]. Partially, this may have been because parents were following the advice of people like Money, who insisted that successful identification with the gender of rearing required that the child be raised to believe in the gender assignment, absolutely. Some parents may have been fearful of divulging any part of the truth, lest it lead to the child failing to identify with the assigned gender.

At a more profound level, it is also likely that parents and physicians fail to divulge the truth of their medical history to intersex children who have been subjected to surgery because of a deep internal discomfort about intersexuality and its implications. Indeed, many of the decisions to go ahead with early surgeries may have more to do with parents’ and physicians’ discomfort with ‘difference’ than with what is truly in the best interests of the child [8]. Cresti et al. write

It seems as though these treatments really ‘have been contrived solely to conform people to our narrow ideas of “normal”…. A reason for this is that human adults are afraid of “atypicality.” They possess specific ideas, culturally situated and socially built, about the kind of body human beings must have, and it is this normativity, imagined by adults, which is incised upon the body of intersexual children’ [15].

Dickens writes:

It was observed 20 years ago in medical practice that parents of intersex children and the children as they mature ‘are lied to; risky procedures are performed without follow-up; consent is not fully informed; autonomy and health are risked because of unproven (and even disproven) fears that atypical anatomy will lead to psychological disaster’ [12].

It is not only intersex persons who have routinely been lied to or have not been fully informed. I have already quoted Greenberg’s assertion that medical experts often believe that it is better for parents to be told ‘less than the whole truth’ [13]. She maintains that physicians do not always inform parents that their child might not end up identifying with the assigned gender after surgery, and that they deliberately downplay revealing anything that might cause confusion, in order that parents will feel comfortable with consenting to surgery [13]. She writes: ‘Although parents believe they are considering the best interests of their children when they make their treatment decision, it is difficult for parents to rationally assess whether they are focusing on their need to have a “normal” infant over the long-term interests of their child’ [13].

None of these reasons for withholding the truth about their conditions and medical history from intersex individuals or their parents can be ethically justified. Again, what is most baffling is the exceptionalism that so often seems to apply in these cases. It is by now an established ethical principle that health professionals ought to tell their patients the whole truth about their health, unless there are very good reasons not to. Clinical medicine rejected paternalism and the so-called physician’s therapeutic privilege to decide what to tell patients decades ago. This generally applies to children, too [12]. Only in cases where telling the truth to a child is likely to be beyond their comprehension can any kind of deception or withholding of the truth be morally justified. And even then, such decisions need to be regularly reviewed in the light of the child’s increasing maturity and understanding. The truth should always be told in an age appropriate manner [12]. These days this is standard practice, even in cases where children have serious illnesses or terminal conditions. We have discovered that children are far more capable of understanding and far more resilient than we used to think. Intersex children should be told the truth just like anyone else. Furthermore, this will contribute to an ending of the conspiracy of silence around intersex and variations of sexual characteristics. Ignorance lies at the root of so much societal prejudice, and silence feeds it.

Infant genital ‘normalisation’ surgeries have been part of the standard approach to intersex births for some decades now, but since the purported grounds for these interventions have been shown to be unfounded, it is becoming more likely that intersex activists, victims of unjustified surgeries, medical experts and society at large will challenge these practices, and the deception that routinely accompanies them. It is likely only a matter of time before someone turns to the courts to challenge these practices. Dickens warns:

However well-intentioned, parents, doctors, psychologists and others risk ethical and legal violations if they seek to escape the challenges of educating parents and providing age-appropriate education to growing children by discarding the ordinary principles of offering and providing medical services, such as honesty, adequate disclosure, and indicated follow- up care [12].

A commitment to truth-telling will also have the consequence of moderating drastic choices by parents and medical teams, as they know they will have to account to the intersex child when she or he is older.

## Principle 5: where physicians and/or parents think that surgery truly is in the best interests of the child, in terms of safety or psycho-social well-being, the burden of proof lies with them

This principle seeks to ensure that the typical or default decision will be to not perform surgery, and that those who want to perform surgery on children will need to provide very good reasons why this is necessary. Given that there are contexts in which intersex infants can be killed or be in constant danger of physical harm, this at least keeps the door open to surgeries performed in the interests of the safety of the child, but only in exceptional cases. What needs to change urgently is resorting to surgery as the default choice. Professional guidelines should clearly recommend delaying surgeries and require physicians and parents who believe that surgery is necessary for the safety of the child to provide a strong motivation for why this is so. It needs to be understood that it is almost always unethical to perform genital ‘normalising’ surgeries on intersex children, where there is no medical necessity. Where such surgeries *are* given the go-ahead, this should be a reluctantly granted concession that is made only because of a serious risk of harm to the child. Furthermore, this should be seen as a last resort, only to be effected when alternatives have been exhausted or where it is thought that they would be ineffectual or impracticable.

To ensure that this concession is not abused, I would also propose that some kind oversight body, comprised of suitably qualified persons, should be required to review applications for early surgeries and approve or reject them on their merits.

## Conclusion

Surgery for intersex infants should be delayed until the intersex individual is able to make their own decisions in this regard, except in cases where surgery is a medical necessity. In an ideal world, this single principle would suffice and surgeries that are not medically necessary could be totally prohibited. Unfortunately, the world is less than perfect, and, in some parts of the world, intersex neonates are at risk of being killed, abandoned or mutilated. These risks of harm accompany some intersex persons throughout their childhood and even into adulthood. As long as intersex persons are at such high risk in some places, any ethical guidelines for intersex surgeries will need to take these extreme risks of harm into account. In this paper, I have therefore argued for a set of five basic principles that can form the foundation for professional ethical guidelines for best practice regarding intersex infants. It is hoped that these principles might help medical teams and parents make better decisions about intersex surgeries on children, and they would make such surgeries very rare indeed, if they happen at all. As Carpenter and Cabral write:

’Normalizing’ procedures violate the right to physical and mental integrity, the right to freedom from torture and medical abuses, the right to not being subjected to experimentation, the right to take informed choices and give informed consent, the right to privacy and, in general, sexual and reproductive rights [18]*.*

I give the last word to South African intersex activist, Nthabiseng Moekwena, who has said:

I am so pleased I never had surgery. The people I met, most of them, black and white, who have had surgery as babies, usually ha[d] confused parents who[m] the doctors incorrect[ly] informed, and the children were subjected to surgery which has ended up being far more traumatic and confusing…

We have been raised in a world that makes us feel like monsters. My advice to other intersex people is to love and accept. Only then will you make the right decision about surgery… Surgery is not a magic pill that has no consequences [19].

## Data Availability

Data sharing is not applicable to this article as no datasets were generated or analysed during the current study.

## Abbreviation

VSC: Variations in sexual characteristics

## Footnotes

1 I deliberately use the term ‘intersex’ throughout this article, as this is appears to be the term preferred by intersex activist groups. There are many Variations in Sex Characteristics (VSC) or Differences in Sex Development, ranging from a minor displacement of the urethral orifice to truly ambiguous genitalia and total discordance of anatomical and chromosomal sex. Many VSCs do not fall into the category of intersex. Since the focus of this paper is on surgeries in infants born with VSCs, the scope of the discussion is limited to those kinds of VSCs that would likely give rise to surgery being considered as a possibility. Because this entails cases where the outward appearance of the genitalia is obviously atypical or ambiguous, the use of the term ‘intersex surgeries’ is appropriate.

2 By contrast, my principles take a stronger position against normalizing surgeries that are not medically necessary than the positions taken in the Chicago Consensus document [8] or the principles proposed by Gillam et al. [20].

3 Many of the kinds of surgeries typically performed on intersex infants are far more than merely cosmetic. They might include castration, hysterectomy, or the creation of an artificial vagina, among others.

4 I use the phrase ‘true medical necessity’ to denote conditions where surgery is required to preserve the life or ensure the physical health of the patient. This would ordinarily exclude surgeries performed solely for the purpose of ‘normalising’ the appearance of the genitalia.

5 Although I argue strongly that ‘normalising’ surgeries should be delayed until children are mature enough to assent to treatment or decide against it, I acknowledge that many parents might consider surgery because they are truly concerned for their children’s well-being. Without access to empirical evidence of the harms that such surgeries can cause, it is understandable that some parents might think that ‘normalisation’ is the best way to protect their child from stigmatisation and growing up ‘different’.

6 The extent to which parents have rights to decide the fate of their children is contentious [21]. However, article 3 of the United Nations Convention on the Rights of the Child states that ‘in all actions concerning children…the best interests of the child shall be a primary consideration’ [22]. In line with this statement, many jurisdictions now recognise, in their law relating to children, that the principle of the best interests of the child should be a paramount consideration. In my own country, South Africa, this principle has been applied to establish the legal precedent that parents’ decisions to deny blood transfusions to children on religious grounds may be overridden and that cultural practices like female circumcision are forbidden. There is a growing consensus position that parents do not have unlimited rights to make decisions on behalf of their children, and that they do not have the right to make decisions that are likely to cause non-trivial, avoidable harm to their children.
